# Low-Burden Oligometastatic Disease of the Lung Treated with Robotic Stereotactic Ablative Radiotherapy: A Retrospective Study

**DOI:** 10.3390/biomedicines13020517

**Published:** 2025-02-19

**Authors:** Anna Zygogianni, Ioannis M. Koukourakis, Zoi Liakouli, Dimitra Desse, Ioannis Georgakopoulos, Christina Armpilia, Georgia Lymperopoulou, Vasileios Kouloulias

**Affiliations:** 1 Radiation Oncology Unit, Aretaieion Hospital, School of Medicine, National and Kapodistrian University of Athens, 11528 Athens, Greece; azygogianni@med.uoa.gr (A.Z.); zliakouli@aretaieio.uoa.gr (Z.L.); ioangeo@med.uoa.gr (I.G.); charbilia@med.uoa.gr (C.A.); glymper@med.uoa.gr (G.L.); 2 Department of Clinical Radiation Oncology, Attikon Hospital, School of Medicine, National and Kapodistrian University of Athens, 12462 Athens, Greece; ikoukourakis@med.uoa.gr (I.M.K.); dimidesse@gmail.com (D.D.)

**Keywords:** lung, oligometastatic disease, cancer, stereotactic radiotherapy

## Abstract

**Background/Objectives**: The lung is the most common site of metastases, regardless of the cancer subtype. Treating oligometastatic disease with surgery or stereotactic ablative radiotherapy (SABR) may improve patient survival. **Methods**: We retrospectively analyzed 41 patients with limited (one or two lesions, max dimension <3 cm) lung-only metastatic disease that were treated with the CK M6 robotic radiosurgery system in our Department, in terms of treatment efficacy and toxicity. **Results**: Acute and late toxicity was negligible (4 out of 41 patients developed grade 2 or 3 lung fibrosis). Six months post-SABR, complete response was achieved in 18 out of 41 patients (43.9%), while the rest of the cases exhibited major responses. A biological effective dose (BED_α/β=10_) in the range of 100 Gy appears to be equally effective with higher doses. Within a median follow-up of 34 months, only three patients (7.3%) progressed locally, while three patients progressed to distal sites. Two-year local progression-free survival (LPFS) rates were 92.6% (95% CI 78.5–97%). **Conclusions**: SABR for low-burden lung oligometastases is an effective treatment modality that yields high local control and survival rates. Toxicity is negligible, regardless of the performance status of patients. Early referral of such patients to radiation oncology departments may be critical for patient survival and quality of life.

## 1. Introduction

Ever since the recognition of cancer as one of the most lethal diseases, scientific and clinical efforts in treating cancer patients have been relentless. Up until the early 2000s, it was a common notion that dissemination of malignant tumors was associated with poor prognosis and eventual death. However, rapid advances in drug development during the past 10 years have significantly prolonged patient survival and, thus, set the beginning of a new era in cancer treatment that strives for complete eradication of metastatic disease [1,2].

Along with the advent of more effective systemic therapy, radiotherapy’s (RT) once determinate role in the definitive treatment of locoregional disease and palliation has now expanded into the radical treatment of limited metastatic disease [3]. The SABR-COMET phase II randomized trial demonstrated that patients with solid tumors and less than five metastatic lesions (lungs, bones, etc.) had significantly longer overall (OS) and progression-free survival (PFS) when standard of care systemic therapy was combined with stereotactic ablative RT (SABR) to the metastatic sites (8-year OS and PFS 27.2% vs. 13.6% and 0% vs. 21.3% in the standard of care + SABR vs. standard of care arms, respectively) [4]. In this context, the European and American Societies for Radiation Oncology (ESTRO and ASTRO) have defined oligometastatic disease as the setting of one to five metastatic lesions that can be safely treated, with primary tumor control being optional [5]. It is also postulated that RT can elicit an effective anti-tumor immune response and systemically contribute to cancer cell killing [6].

The lung is the most common site of metastases, regardless of the cancer subtype [7]. Ablative RT of lung tumors utilizes high irradiation doses per fraction (>8 Gy per fraction for a total of 3–5 fractions), while also sparing the surrounding normal lung parenchyma of significant toxicity. In patients with stage I non-small cell lung cancer (NSCLC), SABR is an acceptable alternative to surgical treatment, with equivalent efficacy, in cases of medical inoperability or patients’ choice not to undergo surgery [8]. Two large retrospective studies investigating SABR for lung oligometastases have showcased local control (LC) rates of >80% for at least 2 years, and >60% 2-year OS [9,10]. Whether robotic stereotactic RT for patients with a limited number of lung metastases can be established as a valuable adjunct to first-line systemic therapy is under investigation.

In this study, and in contrast to previously published data [9,10,11], we focus on patients with lung-only metastatic disease who presented with low tumor burden in terms of number of metastases and tumor size, in order to assess the significance of early detection and therapeutic intervention. We retrospectively analyze a subgroup of patients with such early oligometastatic disease treated with the CK M6 robotic radiosurgery system in our Department in terms of treatment efficacy and toxicity.

## 2. Materials and Methods

This is a retrospective analysis of 41 cancer patients with limited oligometastatic disease (equal to or less than two lesions) of the lung and no other site of metastases. These patients were treated with SABR to the lung lesions via the CK M6 robotic radiosurgery system equipped with the InCise (2) Multileaf Collimator and the LOT module, together with the real-time image-guided Synchrony Respiratory Tracking System (Accuray, Inc., Madison, WI, USA) to deal with target movement, at the Radiation Oncology Unit of Aretaieion University Hospital, Athens, Greece. Patient inclusion criteria comprised histological confirmation of the primary tumor, radiologically (PET/CT or chest CT) or biopsy-confirmed lung-only metastases (1–5 per the ESTRO-ASTRO consensus [5]), peripheral or central lung lesions, and a maximum tumor size equal to or less than 5 cm. The current study, however, analyzes only patients with very low metastatic tumor burden (one or two lesions) measuring less than 3 cm in maximum diameter. This could eventually underscore the significance of early detection of metastatic disease and radiotherapeutic intervention in these cases. A multidisciplinary team consisting of pathologists, radiation and medical oncologists, and thoracic surgeons was appointed to each patient once the diagnosis of metastasis was made, with the goal of deciding on the ideal therapeutic approach. Severe comorbidities (such as cardiovascular and chronic obstructive pulmonary disease) and the patient’s choice to avoid a surgical approach were the main reasons that a metastasectomy was not performed. Systemic therapy, including chemotherapy or immunotherapy, was allowed according to the physician’s discretion. All patients proceeded to receive systemic therapy post-SABR. The median follow-up was 34 months (16–39). Table 1 displays patient and disease characteristics.

Approval of the study was obtained by the local Ethics and Research Committee (approval number no: 484/16-01-2023). All patients gave their written and informed consent before treatment initiation, and agreed on the handling of their treatment data for research purposes.

### 2.1. Planning and Treatment Details

Details regarding treatment planning and delivery have been extensively reported in a previous study concerning patients with NSCLC who were treated with CyberKnife SABR in our Department [12]. For each patient, three series of CT images with 1 mm equidistant slices were acquired (exhalation, inhalation, and normal breathing with IV contrast) using a Philips Brilliance 16 CT-sim scanner. The acquisition included 15–20 cm above and below the tumor, as well as the entire pulmonary volume. Fusion of the acquired CT images with PET/CT was also performed. Gross tumor volume (GTV) was contoured at the exhalation, normal breath, and inhalation phase, and an internal target volume (ITV) was created. No margin was applied for the clinical target volume (CTV) definition. By taking an image of patients on the treatment device, tracking modality was selected according to the visibility of the target (1-View and 2-View). The planning target volume (PTV) was set at 3–5 mm around CTV in the tracked direction and 5–8 mm in the untracked direction. The lungs, spinal cord, ribs, major vessels, heart, esophagus, and major bronchi/trachea were delineated as organs at risk (OAR). Dose constraints for critical organs were used as shown in Table 2 [13,14]. Accuray Precision Treatment Planning software (v.3.1.1.1) was used to generate treatment plans. An InCise2 multileaf collimator optimized by the Monte Carlo algorithm was used for all patients. The prescription dose was prescribed at the 80–90% isodose line with at least 90% coverage of PTV. A pair of orthogonal kV X-ray imaging systems were used for simultaneous target tracking. The CK Xsight Lung Tracking System (fiducial-free motion management), together with the Synchrony Respiratory Tracking System, was utilized for optimal tumor tracking. The mean treatment time for each lung lesion was 21 min per fraction.

SABR schedules varied among patients, based on patient performance status (PS), tumor size, and physician’s choice. The number of RT fractions delivered ranged from 1 to 5 (median 3), while the median dose per fraction was 12 Gy (10–30 Gy). The two most common schedules were 3 × 18 Gy and 5 × 10 Gy.

Using the linear quadratic model, biological effective dose (BED) was calculated according to the following formula: BED= N × d [1 + d/(α/β)], where N is the number of fractions, d is the dose per fraction, and α/β is the ratio characterizing normal and tumor tissues [15]. According to Bentzen et al., for late lung tissue radiation injury, an α/β ratio of 3 Gy was applied [16]. An α/β ratio of 10 Gy was considered for early toxicity. For tumor tissue, the α/β ratio is unknown and potentially different for each primary histology. To serve the needs of the current analysis, an α/β value of 10 Gy was used. The median BED_α/β=10_ delivered to the metastatic lesions was 112.5 Gy. The median BED_α/β=3_ to the lung tissue was 270 Gy. Table 3 shows details on the RT schedules applied.

### 2.2. Treatment Efficacy and Toxicity Evaluation

Daily patient evaluation was performed for potential acute RT sequelae. Patient follow-up consisted of a trimonthly chest CT scan, and PET/CT at the 6-month and 1-year time points post-RT completion, unless there was earlier clinical evidence of disease progression. Local progression-free survival (LPFS) and OS were assessed at each follow-up. LPFS was defined as the time between RT completion and the confirmation of disease progression within the irradiated area, while OS refers to the interval between the end of RT and the last follow-up or death event. Treatment response was determined based on the RECIST and PERCIST criteria at the 3- and 6-month time points, respectively [17,18]. Briefly, local progression is defined as a more than 20% increase in tumor dimensions or a clear increase in FDG uptake in PET images (by more than 30%). Complete disappearance of the irradiated tumor or lack of metabolic activity in PET images characterized complete response (CR). A reduction in tumor size or a decrease in FDG uptake of more than 30% was considered a partial response (PR). Any cases not described by the aforementioned criteria were considered stable disease (SD). CR, PR, or SD constituted LC.

Acute adverse events were scored using the Common Terminology Criteria for Adverse Events (CTCAE) v.5 version [19], while grading of late radiation-induced lung toxicity (RLT) was based on a scale proposed by our group in a previous study [20]. Briefly, grade 0 refers to the lack of any findings. Ground glass opacities refer to grade 1, while the extension of such opacities beyond the irradiation field defined grade 2 lung toxicity. Focal lung tissue consolidation with fibrotic elements characterized grade 3 RLT, while dense consolidation and atelectasis defined grade 4 RLT.

### 2.3. Statistical Analysis

The GraphPad Prism 8.0.2 version was used for statistical analysis and graph presentation of the Kaplan–Meier survival curves. Using univariate and multivariate regression models that were performed through IBM SPSS Statistics Version 26, we assessed the impact of BED_α/β=10_, volume, systemic treatment, and histology on tumor response. A *p*-value < 0.05 was considered for statistical significance.

## 3. Results

Eighty-three percent (83%) of the patients displayed a good PS of 0 or 1. The median patient age was 72 years. The most frequent source of lung metastasis was NSCLC and breast carcinomas. Seventy five percent of patients (75%) had a solitary metastasis, with the remaining having two documented lung lesions. All patients had peripheral tumors, measuring less than 3 cm at maximum diameter and a maximum volume of 29 cc (Table 1).

### 3.1. Toxicity

No signs of acute lung toxicity were recorded in our patients. As far as late lung toxicity is concerned, four (9.7%) patients developed grade 2 or 3 RLT. There were no cases of acute or late adverse events from the skin, vessels, esophagus, or chest wall.

### 3.2. Response

**Figure 1:**
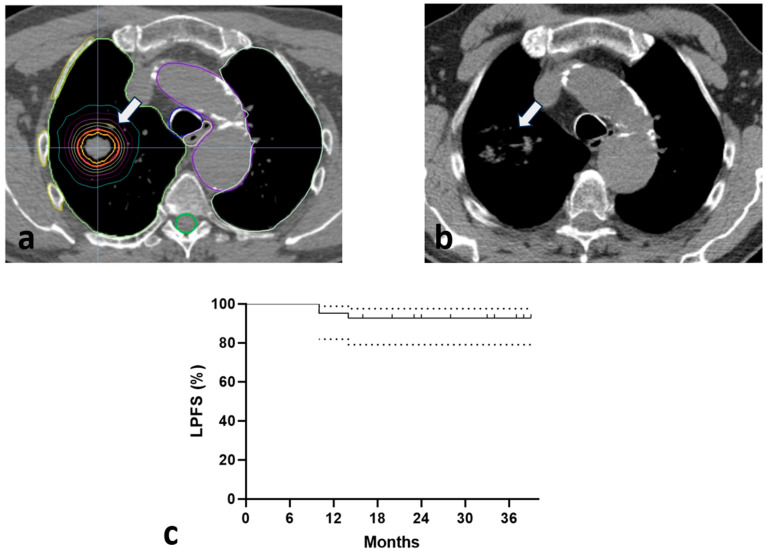
A typical example of a complete response (white arrows) of a lung metastasis 6 months after radiotherapy (colored circular lines around the tumor display the isodose curves; all other lines concern organs at risk) (**a**,**b**). Kaplan–Meier locoregional progression-free survival (LPFS) curves [Dotted lines show the 95% confidence intervals (CI)] (**c**).

Six months post-SABR, radiological examination confirmed the complete disappearance of the lesions in 18 out of 41 patients (43.9%), while all the remaining patients (56.1%) exhibited major responses in the range of 70% tumor reduction and above, in parallel with a significant reduction in SUVmax. Thus, LC was achieved in 100% of patients. Figure 1a,b show a typical example of a CR 6 months after irradiation. As shown in Table 4, only primary histology had an impact on response rate, either in univariate or multivariate analysis.

### 3.3. Survival

Within a median follow-up of 34 months, four patients died from intercurrent disease. None of the patients died from cancer. Only three (7.3%) patients progressed locally, while three patients presented with distant metastases. Overall, 3-year progression-free survival rates were 87.8% (95% CI 78–97%). Figure 1c displays the Kaplan–Meier LPFS curves (3-year LPFS 92.6%, 95% CI 78.5–97%).

Table 5 highlights the results of SABR.

## 4. Discussion

Due to the high radiosensitivity of the lung tissue and the clinically severe damage induced to the lungs through high doses and extensive irradiation fields, the use of RT in the treatment of lung metastases has not been widely applied, with the exception of in extremely radiosensitive tumors like Ewing sarcoma [21]. RT in patients with limited lung metastases can be, however, safely applied. In a study by Fleming et al. [22], 99 patients were treated with conventionally fractionated RT, showing efficacy in the alleviation of symptoms and acceptable 1-year LC rates of approximately 57%. Interestingly, comparison with a cohort of 91 patients treated with stereotactic body radiation therapy (SBRT) revealed increased 1-year LC rates (81%). During the past decade and following the rapid evolution of stereotactic techniques, SBRT has been gradually adopted in the standard clinical practice as an effective tool to eradicate lung lesions, especially in the oligometastatic setting.

Several randomized trials have displayed that stereotactic RT to limited metastatic disease is associated with an improvement in PFS and/or OS [4,23,24]. As far as pulmonary oligometastases is concerned, in 2010, a systematic review on stereotactic RT for metastatic lung lesions reported 2-year LC rates of 78% with limited toxicity (3–4% grade 3 or higher). More than 50% of patients were alive at 2 years [25]. A retrospective study on 700 patients with pulmonary metastases treated with SBRT reported 2-year LC rates as high as 81.2% and 2-year OS rates of 54.4%. Median dose per fraction was 12.5 Gy and grade 2 or higher RLT was noted in 6.5% of patients. BED was significantly linked with LC [26]. More recently, Niibe et al. reported on 1378 patients that received SABR for lung oligometastases with a BED_α/β=10_ of more than 75 Gy [9]; 3-year OS was >60%, while grade 5 toxicity occurred in three patients. In a series of 301 patients with NSCLC and metastatic lung lesions, 2-year LC and distant control rates were 82% and 45%, respectively [10]. Favorable prognostic factors for LC included histologic subtype (adenocarcinoma over squamous histology) and higher BED at the target isocenter. In addition, Ricco et al. displayed that survival rates varied among patients with different primaries that underwent SBRT for lung metastases; 3-year LC rates reached 77%. A higher BED_α/β=10_ (>100 Gy) was also shown to dictate LC significantly [11].

In our study, within a median follow-up of 34 months, only three patients had progressed locally, while most patients were alive and free of disease progression at the time of the last follow-up. Treatment toxicity was also minimal. No significant correlations were revealed due to the limited number of progression and death events, which can be attributed to the early referral of patients to our Department once the diagnosis of metastases was made and the very low metastatic tumor burden (one or two relatively small lesions; the majority of patients had only one lesion). This is in accordance with the findings of Rieber et al., confirming that OS was significantly influenced by the number of metastases and tumor size [26]. Ricardi et al. also found that a volume of less than 30 cc was linked with a better prognosis [27]. In contrast to the aforementioned trials, we found no statistically significant association between BED or primary histology and LC. This could be due to the fact that 83% of the patients received a high BED (median dose >112.5 Gy), which would suggest higher LC rates as proposed by previous studies [9,10,26]. In addition, the higher BED prescribed could also ignore the radiosensitivity of each histology to a certain point and confer similar results in all cases. In a critical review by Pacifico et al., the authors suggested that LC appears to not be defined by primary histology, although certain studies have suggested colorectal cancer metastases to be more radio-resistant [3]. It is notable that in our study, the extent of treatment response appeared to be linked with the histology of the primary tumor. Thus, radiosensitivity could still play an important role when it comes to RT response even after ablative doses; future trials will eventually determine the appropriate regimen for metastatic patients with different primary histology. In this context, cancer stem cells, known to be responsible for tumor proliferation, growth, and metastasis, have been shown to be an important factor contributing to tumor chemo- and radio-resistance [28,29]. Recent advances in the field have focused on novel biomarkers for cancer stem cell identification, such as Lgr5 (a G-protein-coupled receptor), that could eventually be targeted in an attempt to reverse this innate resistance of tumors to chemotherapy and RT [30]. The delivery of high dose RT, together with such targeted therapies, appears to be a promising approach that could prevent tumor recurrence and metastasis through cancer stem cell killing. Standard RT techniques, however, are incapable of delivering ablative doses without compromising the surrounding normal tissues [31]. In this respect, SABR could also serve this purpose, especially in cases presenting with limited (in number and size) oligometastases.

There are limited data comparing metastasectomy to SBRT for metastatic lung lesions. Lee et al. displayed better PFS and OS for 30 patients undergoing metastasectomy of the lung when compared to 21 patients that underwent SBRT (2-year OS 81.8% vs. 68.2%; 2-year PFS 46% vs. 11.9%). Nevertheless, this difference was erased when patients were divided according to the presence of other synchronous metastases, which was shown to be a predictive factor for poor PFS. In fact, SBRT was used more frequently for these patients [32]. Garcia-Exposito et al. analyzed 75 colorectal cancer patients with lung metastases treated with either SBRT or surgery [33]. There was no significant difference in terms of 2-year LPFS (71% for SBRT and 70% for surgery, respectively). A more recent retrospective analysis of 251 patients with colorectal cancer also displayed better PFS for patients undergoing metastasectomy; however, OS was similar between groups (5-year 73.1% vs. 68.7%) [34]. Overall, SBRT is an effective, non-invasive approach that can safely be used for the treatment of more than one metastases, sparing patients from extensive surgical procedures and subsequent complications. Moreover, multiple experimental data have demonstrated that RT has a significant interplay with the patient’s immune system and can potentially lead to systemic enhancement of anti-tumor immunity [6]. This suggests another advantage of RT over surgery in the metastatic setting, as SABR’s efficacy is not field-restricted, but can also activate important immune pathways, such as the IFN-type-I response, and confer abscopal sequelae [35,36]. In a recent phase 2 randomized trial, it was displayed that the combination of SABR with nivolumab for early-stage NSCLC leads to significantly higher 4-year event-free survival rates when compared to SABR alone (77% vs. 53%), further supporting the synergistic effects of RT with immunotherapy [37].

This study has certain limitations. A larger sample size would allow us to extract safer conclusions regarding the effect of SABR on patients with lung oligometastases and define predictors of LC, PFS, and OS. The relatively short follow-up of 36 months is another drawback of our study that hampers the evaluation of long-term outcomes; however, the encouraging results provided—given the metastatic status of the patients—can hopefully contribute to the initiation of larger trials that will strive to address the significance of SABR in patients with lung only low-burden oligometastatic disease. The limited number of progression or death events further hinders extensive analysis, although this could be attributed to the early referral of low-burden oligometastatic patients to our Department who were previously successfully treated for their primary disease. Moreover, the treatment decision being based on PET/CT rather than histological confirmation in the majority of patients, as a result of the very small lung lesions, is another limitation.

## 5. Conclusions

SABR for low-burden lung oligometastases is an effective treatment modality that yields high local control and survival rates. Toxicity is negligible, regardless of the PS of patients, and SABR can be administered with minimal discomfort (one to three hospital visits). The lack of invasiveness is an important advantage over surgical metastasectomy. Inclusion of SABR as an early step in the treatment algorithm of patients with limited lung metastatic disease, and early referral of such patients to radiation oncology departments immediately after the onset of systemic therapy or at least after documentation of incomplete response may be critical for patient survival and quality of life. Whether SBRT combination with novel immunotherapy agents—given the postulated immuno-stimulatory properties of SBRT—can further improve the prognosis of patients with oligometastatic lung disease is a hypothesis that should be examined in future trials.

## Figures and Tables

**Table 1:** Patient and disease characteristics.

No pts	41	%
PS		
0	15	36.6
1	19	46.4
2	6	14.6
3	1	2.4
Gender		
Male	19	46.4
Female	22	53.6
Age		
Median	72	
Range	40–84	
Primary tumor		
Breast cancer	10	24.5
Colorectal cancer	8	19.5
Head and neck cancer	1	2.4
Non-small cell lung cancer	14	34.1
Sarcoma	7	17.1
Gastric cancer	1	2.4
Number of lung metastases treated (total)	51	
1 (per patient)	31	75.6%
2 (per patient)	10	24.4%
Size (mm)		
Median	17	
Range	7–30	
33rd Percentile	16	
66th Percentile	18	
Volume (cc)		
Median	22	
Range	3–29	
33rd Percentile	19	
66th Percentile	23	
Location		
Peripheral	51	100%
Concurrent systemic therapy		
None	3	7.3
Chemotherapy	20	48.7
Immunotherapy ± Chemo	18	44

**Table 2:** Dose constraints for critical organs at risk.

Critical Structure	Max Critical Volume Above Threshold	Threshold Dose (Gy)	Max Point Dose (Gy)
Spinal cord	<0.35 cc	18	21.9
Esophagus	<5 cc	17.7	25.2
Heart	<15 cc	24	30
Great vessels	<10 cc	39	45
Trachea and large bronchus	<4 cc	15	30
Lungs (Right and Left)	<1500 cc	10.5 Gy	-
Rib	<1 cc	28.8	36.9
	<30 cc	30	-

**Table 3:** Radiotherapy schedules applied and biological effective dose (BED).

Fractions/ Dose Per Fraction	No. Patients	BED_α/β=3_ (Gy)	BED_α/β=10_ (Gy)
1/30 Gy	1	330	120
3/20 Gy	1	460	180
3/18 Gy	8	378	151.2
3/17 Gy	5	340	137.7
3/15 Gy	5	270	112.5
3/12 Gy	6	180	79.2
5/11 Gy	3	256.7	115.5
5/10 Gy	11	216.7	100
15/3.5 Gy	1	113.5	70.9

**Table 4:** Univariate and multivariate regression analysis for the impact of BED_α/β=10_, volume, systemic treatment and histology on tumor response. (F = 3.12, P = 0.04).

	Univariate		Multivariate	
Covariate	R (95% CI)	P	R (95% CI)	P
Primary	0.23 (0.11–0.72)	0.018	0.44(0.21–0.67)	0.012
Systemic treatment	0.05	0.78		
Volume	−0.29	0.073	-	
BED_α/β=10_	0.33	0.091	-	

**Table 5:** Toxicity, response, and survival following stereotactic ablative radiotherapyfor patients with limited oligometastatic disease.

No pts	41	%
Lung Toxicitiy		
Early	0	0
Late		
Grade 0	12	29.3
Grade 1	25	61
Grade 2	3	7.3
Grade 3	1	2.4
Response		
Complete response	18	43.9%
Partial response (>70%)	23	56.1%
Survival rates (3-year)		
Overall survival	37	90.2%
Local progression-free survival	38	92.6%
Metastasis-free survival	38	92.6%
Progression-free survival	36	87.8%

## Data Availability

Research data are stored in an institutional repository and will be shared upon reasonable request to the corresponding author.
